# Screening of 20 species from Lamiaceae family based on phytochemical analysis, antioxidant activity and HPLC profiling

**DOI:** 10.1038/s41598-023-44337-7

**Published:** 2023-10-09

**Authors:** Atefeh Moshari-Nasirkandi, Abolfazl Alirezalu, Hadi Alipour, Jussara Amato

**Affiliations:** 1 Department of Horticultural Sciences, Faculty of Agriculture, Urmia University, Urmia, Iran; 2 Department of Plant Production and Genetics, Urmia University, Urmia, Iran; 3 Department of Pharmacy, University of Naples Federico II, Naples, Italy

**Keywords:** Chemical biology, Plant sciences

## Abstract

The Lamiaceae family encompasses numerous species highly valued for their applications in medicine, food, and cosmetics. In order to screen the Lamiaceae family and discover new sources of phytochemicals and antioxidants, we comprehensively evaluated 20 species from this family, including *Phlomis herba-venti*, *P. tuberosa*, *P. olivieri*, *P. kurdica*, *Nepeta* sp.,* N. cataria*, *N. saccharata*, *Stachys* sp., *S. inflata*, *Scutellaria albida*, *Marrubium parviflora*, *Mentha pulegium*, *Thymus kotschyanus*, *Lamium album*, *Salvia officinalis*, *S. multicaulis*, *S. macrochlamys*, *S. candidissima*, *S. verticillata*, and *S. nemorosa*. The aerial parts of these species were analyzed to determine their total phenolic (TPC) and flavonoid (TFC) contents, total tannin content (TTC), ascorbic acid content (AAC), antioxidant capacity (assessed by FRAP and DPPH assays), and polyphenolic components (by HPLC). The phytochemical compounds and antioxidant properties varied widely among different species. The highest concentrations of TPC (70.93 mg GAE/g DW), TFC (17.89 mg Que/g DW), TTC (6.49 mg TAE/100 g), and AAC (1.15 mg AA/g DW), as well as the greatest antioxidant activity, were observed in different *Salvia* species. Additionally, chlorogenic and rosmarinic acids were the primary phenolic compounds identified in the extracts from the investigated Lamiaceae family. According to Hierarchical Cluster Analysis (HCA) and Principal Component Analysis (PCA), three groups of species were identified, characterized by variations in phytochemical composition and antioxidant capacity. The results obtained can provide new natural sources of phytochemicals and antioxidant agents, particularly from *Salvia* species, for the advancement of new products in the food, agricultural, cosmetics and health industries.

## Introduction

The Lamiaceae family, also known as the mint family, is a diverse group consisting of approximately 230 genera and 7100 species found worldwide. This family holds significant importance due to its numerous applications in medicine, culinary arts, and cosmetics^1^. In Iran, the Lamiaceae family exhibits remarkable diversity and distribution, comprising 46 genera and 410 species and subspecies. From these 410 species, 124 species and subspecies (30%) are unique to Iran, making them endemic. Some notable genera within the Lamiaceae family in Iran include *Nepeta* (76 species), *Salvia* (56 species), *Stachys* (34 species), *Scutellaria* (19 species), *Phlomis* (17 species), *Eremostachys* (16 species), *Thymus* (16 species), and *Teucrium* (12 species). Additionally, some of the largest genera within the Lamiaceae family are *Thymus*, *Rosmarinus*, *Mentha*, *Salvia*, *Melissa*, and *Origanum*^2^. These genera are known for their diverse range of biological activities and contain a wide variety of phytochemicals. The major bioactive constituents found in these commonly encountered Lamiaceae species include volatile terpenoids, essential oils, hydroxycinnamic acids, phenolic acids, and flavonoids, which display diverse biological activities^3^. Phenolic compounds constitute a group of phytochemical substances that function as secondary metabolites in various plants. Hydroxycinnamic acids (HAs) such as rosmarinic (RS), ferulic (FE), caffeic (CA) and coumaric (CU) acids are important natural antioxidant and phenolic compounds. They have attracted general interest due to their potential positive effects on human health, including antioxidant, anti-inflammatory activities, regeneration, anticatarrhal effects, neuroprotective actions, antibacterial, antiviral, antidepressant, anticancer, antidiabetic, antiangiogenic, antihepatotoxic properties and more^4–8^. The presence of these compounds in wild or traditional plants has garnered increased attention due to their ability to scavenge reactive oxygen species (ROS). As a result, they are highly valued for their potential incorporation into daily diets to enhance overall health and well-being^9,10^. A study conducted on seventy taxa of the Lamiaceae family revealed that numerous species exhibited DPPH radical scavenging activity. Specifically, in four Lamiaceae plants, including *Melissa officinalis*, the DPPH radical scavenging activity was found to be correlated with the content of rosmarinic acid and its derivatives^11^. This suggests that the presence of rosmarinic acid and its derivatives in these plants plays a role in their capability to scavenge DPPH radicals^12^.

With the advancement of modern medicine and pharmaceutical research, chemical synthesis has emerged as the primary method for producing medicinal agents in industrialized countries. However, in developing countries where a significant portion of the world’s population cannot afford pharmaceutical drugs, reliance continues to be made on traditional indigenous herbal medicines. In addition, traditional medicinal plants have garnered considerable attention in the field of drug discovery through the identification and study of their bioactive components. In this context, plants of the Lamiaceae family are of great importance offering potential avenues for the development of new therapeutic agents. Herein, with the aim of screening the Lamiaceae family and discover new sources of phytochemicals and antioxidants, 20 species of this family were comprehensively evaluated.

## Materials and methods

### Collection of plant samples

**Figure 1:**
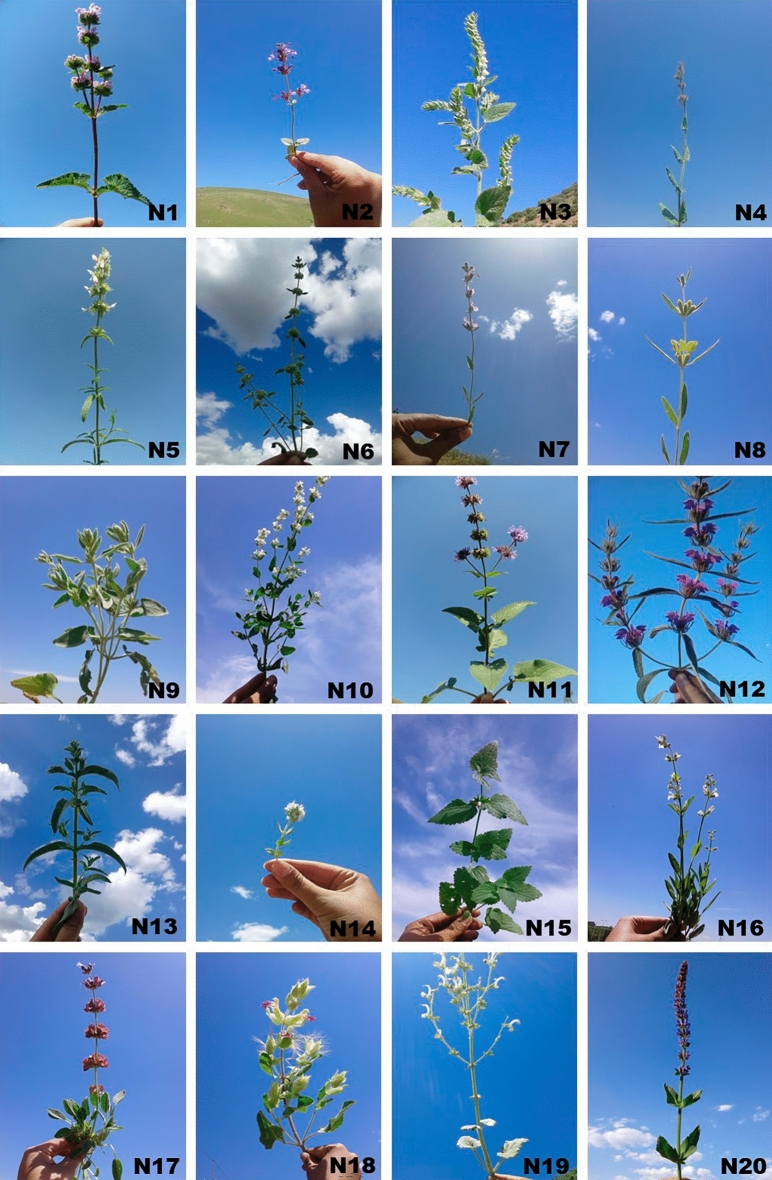
Pictures of the 20 species within the Lamiaceae family investigated in this study.

Aerial parts of various Lamiaceae species (Table 1; including 20 species) during their full flowering phase were harvested from different habitats of the West Azerbaijan province, Iran, in June and July 2020. The species identification of various Lamiaceae samples was conducted at Urmia University by botanist Dr. Bahadori (Fig. 1).

**Table 1:** List of the 20 species investigated within the Lamiaceae family and their sampling locations.

Code	Species	Sampling locations	Height	Longitude	Latitude
N1	*Phlomis tuberosa*	Chaldoran	1886	44°23′04	39°02′40
N2	*Nepeta saccharata*	Ag bulag	1871	46°37′03	36°59′27
N3	*Scutellaria albida*	Marmisho	1741	44°37′42	37°34′44
N4	*Nepeta* sp	Qushchi	1801	44°57′11	38°00′46
N5	*Stachys* sp	Chaldoran	2383	44°17′15	39°01′57
N6	*Marrubium parviflora*	Qushchi	1808	44°56′19	38°00′17
N7	*Stachys inflata*	Saqqez	1498	46°11′34	36°23′43
N8	*Phlomis olivieri*	Qoshachay	1745	46°38′31	36°58′04
N9	*Phlomis kurdica*	Saqqez	1479	46°11′39	36°23′36
N10	*Nepeta cataria*	Piranshahr-Sardasht Rd	1451	45°17′14	36°29′36
N11	*Salvia verticillata*	Mavana	1648	44°47′24	37°33′41
N12	*Phlomis herba-venti*	Hesarlu	1745	46°38′31	36°58′04
N13	*Mentha pulegium*	Xan-darasi	1437	45°07′18	37°17′57
N14	*Thymus kotschyanus*	Qoshachay	1757	46°38′30	36°58′10
N15	*Lamium album*	Pardanan	1453	45°17′14	36°29′36
N16	*Salvia officinalis*	Urmia University	1369	44°58′38	37°39′23
N17	*Salvia multicaulis*	Hesarlu	1745	46°38′31	36°58′04
N18	*Salvia macrochlamys*	Marmisho	1576	44°42′50	37°37′06
N19	*Salvia candidissima*	Marmisho	1703	44°38′56	37°35′09
N20	*Salvia nemorosa*	Serow	1682	44°55′13	37°43′43

### Preparation of methanolic extracts

Dried aerial parts of each Lamiaceae species (1 g) were pulverized individually using liquid nitrogen and then extracted with methanol/water (80%, v/v) using ultrasound-assisted extraction (Elmasonic, Germany) at 30 °C for 30 min. The resulting extracts were stored at 4 ˚C for further parameter measurements.

### Total phenolic content (TPC)

The Folin–Ciocalteu method, slightly modified according to Slinkard and Singleton^13^, was employed to determine the total phenol concentration in the extracts from the Lamiaceae family^13^. Specifically, 1 mL of diluted (1:10) Folin–Ciocalteu reagent (FCR) was mixed with 5 μL of the extract, followed by addition of 480 μL of sodium carbonate (7.5 g in 100 mL) and the resulting mixture was allowed to rest for 5–10 min at room temperature. Subsequently, a spectrophotometer (UNICO, China) was used to measure the absorbance of the samples after a 30 min incubation at room temperature in the darkness. The results were expressed as milligram gallic acid equivalents per gram of dry weight of sample (mg GAE/g DW) using the gallic acid standard curve.

### Total flavonoid content (TFC)

The aluminum chloride colorimetric method proposed by Chang et al. was employed, with slight modifications, to determine the flavonoid content of all extracts^14^. To accomplish this, 200 μL of 5% sodium nitrite and 300 μL of 10% ammonium chloride were added to 5 μL of methanolic extract and stirred for 5 min. After this period, 0.2 mL of 1 M NaOH and 5 mL of distilled water were subsequently added. The absorbance of each sample was measured at 415 nm after a 40 min incubation at room temperature. The results of the TFC were expressed as mg of quercetin equivalent per gram of dry weight sample (mg Que/g DW) employing the quercetin standard curve.

### Total tannin content (TTC)

The TTC was determined according to the method of Bharath et al.^15^. A reaction mixture consisting of 0.1 mL of extract, 2 mL of 4% vanillin solution in MeOH, and 1 mL of hydrochloric acid (concentrated grade) was prepared. The reaction mixture was thoroughly shaken and then incubated at 30 °C for 30 min. The absorbance of each sample was measured at 500 nm. The TTC was expressed as mg tannic acid equivalent per 100 g dry weight sample (mg TAE/100 g DW).

### Phenolic composition analysis by liquid chromatography (HPLC)

In the current study, the major phenolic composition of various Lamiaceae species consisting of gallic acid, *p*-hydroxybenzoic acid, chlorogenic acid, *p*-coumaric acid, caffeic acid, ferulic acid, rosmarinic acid, rutin, hyperoside, luteolin, apigenin was evaluated by HPLC. The equipment comprised an integrated system with a degasser (Smartline manager 5000), pump (Knauer, Smartline system 1000, Berlin, Germany), auto-sampler (Jasco AS-2057), and UV detector. The specifications of the analytical column and the elution program used for the analysis are provided in Table 2. Data acquisition and integration were carried out using the EZChrome Elite software^16^.

**Table 2:** Specifications of the analytical column and elution program used for the analysis of phenolic compounds.

(a)				
Program time	45.01 min			
Flow rate	0.7 mL/min			
Column temperature	35 °C			
Injection volume	20 μl			
Solvent A	H_2_O with 0.1% formic acid			
Solvent B	acetonitrile			
Column	Prontosil 120-5-C18 SH, 250 × 4.6 mm, 5 μm			

(b)				
Time (mins)		Modules	Command	Conc. B
1	0.01	Controller	Start	
2	01.00	Pump	Pump B	20%
3	20.00	Pump	Pump B	30%
4	28.00	Pump	Pump B	98%
5	35.00	Pump	Pump B	98%
6	35.01	Pump	Pump B	20%
7	45.00	Pump	Pump B	20%
8	45.01	Controller	Stop	

### Ascorbic acid content (AAC)

The AAC was evaluated using the method reported by Klein and Perry^17^. Briefly, a sample powder (0.1 g) was extracted using metaphosphoric acid (1% HPO_3_, 3 mL) for 35 min at 4 °C and then filtered. The filtrate was mixed with dichlorophenolindophenol (DCPIP, 2 mL) and the absorbance was recorded within 30 min at 520 nm against a blank. The results were presented as milligrams of ascorbic acid per gram of dry weight sample (mg AA/g DW).

### Antioxidant activity by 2, 2′-diphenyl-1-picrylhydrazyl (DPPH) assay

The free radical scavenging capacity was assessed using the DPPH radical elimination method according to the procedure of Shimada et al., with some modifications^18^. Different concentrations of the extracts were combined with 4 mL of a DPPH methanol solution (0.004%). The reaction mixture was thoroughly shaken and then incubated for 30 min at room temperature in darkness. Subsequently, absorbance was measured at 517 nm. The antioxidant capacity was determined according to the following equation:

$$Inhibition \, \left( \% \right) \, = \, \left( {1 \, - \, absorbance \, \;of\; \, the \, \;sample/absorbance\; \, of\; \, the \, \;blank} \right) \, \times \, 100$$

The DPPH scavenging activity of the samples was expressed as milligrams of ascorbic acid equivalents (AAE) per g of dry weight sample (mg AAE/g DW).

### Antioxidant activity by FRAP assay

The antioxidant potential of the methanolic extract from various Lamiaceae species was assessed using the ferric reducing antioxidant power method (FRAP), as described by Miao et al.^19^. This was achieved by diluting 900 μL of fresh FRAP reagent (prepared by mixing 2.5 mL of 10 mM TPTZ solution in 40 mM HCl, 25 mL of 0.3 M acetate buffer (pH 3.6), and 2.5 mL of 20 mM FeCl_3_^.^6H_2_O with a specific volume of methanolic extract. The resulting mixture was then incubated at 36 °C using a water bath. After incubation, ferric reducing ability of plant extracts was measured at 593 nm. The FeSO_4_·7H_2_O was used for building the calibration curve, and results are expressed as μmol Fe^2+^ per g of dry weight sample (μmol Fe^2+^/ g DW).

### Statistical analysis and screening of various Lamiaceae species

The obtained data were analyzed based on one-way ANOVA (with three replications) using SAS software version 9.4 (SAS Institute Inc., Cary, NC, USA)^20^. Correlation coefficients heatmap plot based on Pearson’s method was created using corrplot R-package. Hierarchical cluster analysis (HCA) was performed using Ward's method^21^ with Euclidean distance algorithm in gplots R-package. Principal components analysis (PCA)^21^ was carried out using factoextra package in R 4.1.0 software^22^.

### Guideline statement

Authors confirm that the use of plants in the present study complies with international, national and/or institutional guidelines.

## Results and discussion

### Phenolic phytochemicals of different Lamiaceae species (TPC, TFC, and TTC)

The TPC, TFC and TTC contents for the different Lamiaceae species are reported in Table 3. Significant differences in TPC, TFC and TTC were observed among the samples (*p* < 0. 01). In particular, TPC content spanned from 21.28 to 70.93 mg GAE/g DW, with *Salvia multicaulis* and *Nepeta* sp*.* exhibiting the highest and lowest TPC values, respectively. On the other hand, the TFC content ranged from 1.85 to 17.89 mg Que/g DW, with *Salvia nemorosa* showing the highest content of total flavonoids and *Nepeta* sp the lowest content. As for the TTC content, it ranged from 0.33 to 6.49 mg TAE/100 g DW, with *Salvia macrochlamys* and *S. verticillata* showing the highest and lowest values, respectively. Collectively, these results suggest that the different Lamiaceae species analyzed are a large source of phenolic phytochemicals. Among these, those belonging to the genus *Salvia* are overall more abundant in TPC, TFC and TTC than the other species, while those of the genus *Nepeta* together with the species *Lamium album* are the poorest. The following descending order was observed for TPC in the different Lamiaceae species: *S. multicaulis* > *S. nemorosa* > *S. macrochlamys* > *S. verticillata* > *S. officinalis* > *S. candidissima* > *Stachys inflata* > *Phlomis olivieri* > *Marrubium parviflora* > *Thymus kotschyanus* > *Phlomis tuberosa* > *Stachys* sp*.* > *Phlomis kurdica* > *Mentha pulegium* > *Scutellaria albida* > *Nepeta saccharata* > *Nepeta cataria* > *Lamium album* > *Phlomis herba-venti* > *Nepeta* sp*.* Although there is a high variation in phenolic compounds across different species, it is also well known that concentration of phytochemicals (including TPC, TFC and TTC) in herbs depend not only on genetics (plant species), but can also vary depending on the climatic conditions, ecological factors, growing location, developmental stage^23^, and methods used for extraction and/or calibration^24^. Indeed, several features, including weather conditions (light intensity, temperature, precipitations, and relative humidity), geographic coordinates (altitude, longitude, and latitude), soil conditions, and genetic factors have been found to contribute to the differences in the amount of phytochemicals across the herbs^25^. Phenolic compounds belong to the largest class of phytochemicals in medicinal herbs. They represent a large group of secondary metabolites in Lamiaceae family with a wide range of biological and chemical actions^26^.

**Table 3:** Phytochemical contents and antioxidant activity for the 20 investigated species within the Lamiaceae family.

Species	TPC (mg GAE/g DW)	TFC (mg Que/g DW)	TTC (mg TAE/100 g DW)	AAC (mg AA/g DW)	AOA by DPPH assay (mg AAE/g DW)	AOA by FRAP assay (μmol Fe^2+^/g DW)
*Phlomis tuberosa*	25.14 ± 0.26	7.97 ± 0.03	0.92 ± 0.02	0.93 ± 0.03	42.76 ± 2.99	16.93 ± 0.27
*Nepeta saccharata*	22.78 ± 0.81	2.99 ± 0.14	1.95 ± 0.09	0.98 ± 0.12	31.47 ± 1.70	16.96 ± 0.05
*Scutellaria albida*	23.54 ± 0.19	5.24 ± 0.08	1.14 ± 0.01	0.63 ± 0.12	31.93 ± 1.02	18.73 ± 0.27
*Nepeta* sp.	21.28 ± 0.31	1.85 ± 0.05	1.24 ± 0.17	0.81 ± 0.06	26.67 ± 0.22	3.76 ± 0.51
*Stachys* sp.	24.95 ± 0.75	11.49 ± 0.35	0.62 ± 0.01	0.58 ± 0.02	45.15 ± 2.30	16.31 ± 0.24
*Marrubium parviflora*	25.73 ± 0.36	10.99 ± 0.26	1.65 ± 0.09	0.82 ± 0.03	48.29 ± 1.97	30.66 ± 0.45
*Stachys inflata*	26.30 ± 0.37	13.71 ± 0.17	1.91 ± 0.01	0.69 ± 0.07	49.41 ± 2.48	25.56 ± 0.44
*Phlomis olivieri*	25.78 ± 0.18	16.36 ± 0.83	2.43 ± 0.02	0.85 ± 0.07	48.65 ± 0.41	27.47 ± 0.12
*Phlomis kurdica*	24.24 ± 0.43	14.22 ± 0.40	2.16 ± 0.01	0.88 ± 0.01	48.97 ± 1.03	28.8 ± 0.20
*Nepeta cataria*	22.56 ± 0.40	3.24 ± 0.38	0.74 ± 0.03	0.97 ± 0.01	47.19 ± 1.81	15.92 ± 0.53
*Salvia verticillata*	32.61 ± 0.39	11.32 ± 0.23	0.33 ± 0.07	0.96 ± 0.03	58.05 ± 3.31	41.38 ± 1.62
*Phlomis herba-venti*	22.14 ± 0.64	9.71 ± 0.29	0.53 ± 0.01	0.9 ± 0.08	36.25 ± 2.45	15.62 ± 0.70
*Mentha pulegium*	23.86 ± 0.31	7.30 ± 0.25	1.05 ± 0.09	0.92 ± 0.12	49.35 ± 2.96	23.29 ± 0.71
*Thymus kotschyanus*	25.42 ± 0.15	9.93 ± 0.31	0.86 ± 0.07	0.91 ± 0.14	51.46 ± 2.18	30.66 ± 2.99
*Lamium album*	22.32 ± 0.56	2.54 ± 0.06	0.78 ± 0.08	0.93 ± 0.16	27.33 ± 0.67	13.36 ± 0.70
*Salvia officinalis*	31.32 ± 0.68	10.36 ± 0.75	3.96 ± 0.17	1.15 ± 0.02	56.30 ± 3.31	56.14 ± 1.86
*Salvia multicaulis*	70.93 ± 0.16	15.57 ± 0.66	4.64 ± 0.25	1.02 ± 0.08	56.85 ± 3.91	67.58 ± 2.09
*Salvia macrochlamys*	34.67 ± 0.33	11.91 ± 0.09	6.49 ± 0.06	0.93 ± 0.07	58.78 ± 2.69	77.21 ± 1.79
*Salvia candidissima*	28.35 ± 0.13	10.44 ± 0.04	4.99 ± 0.66	1.1 ± 0.05	54.62 ± 3.46	62.02 ± 1.11
*Salvia nemorosa*	45.50 ± 0.50	17.89 ± 0.20	4.31 ± 0.33	0.83 ± 0.03	58.86 ± 3.91	62.47 ± 1.06

*TPC* Total phenolic content, *TFC* Total flavonoid content, *TTC* Total tannin content, *AAC* Ascorbic acid content, *AOA* antioxidant activity.

### HPLC profiling of phenolic phytochemicals

**Figure 2:**
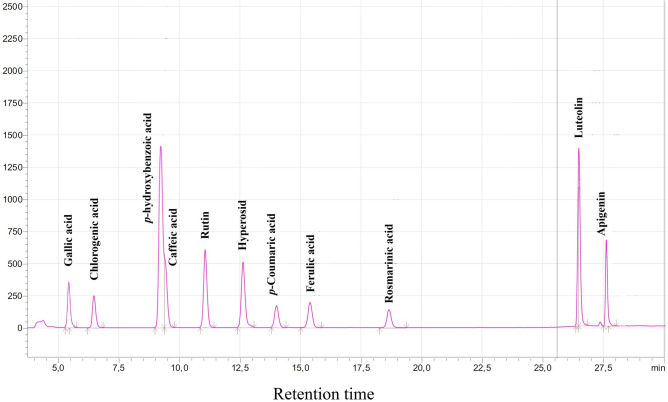
HPLC chromatograms of standard solutions of phenolic compounds (Gallic acid, Chlorogenic acid, *p*-hydroxybenzoic acid, Caffeic acid, Rutin, Hyperoside, *p*-Coumaric acid, Ferulic acid, Rosmarinic acid, Luteolin, and Apigenin).

**Figure 3:**
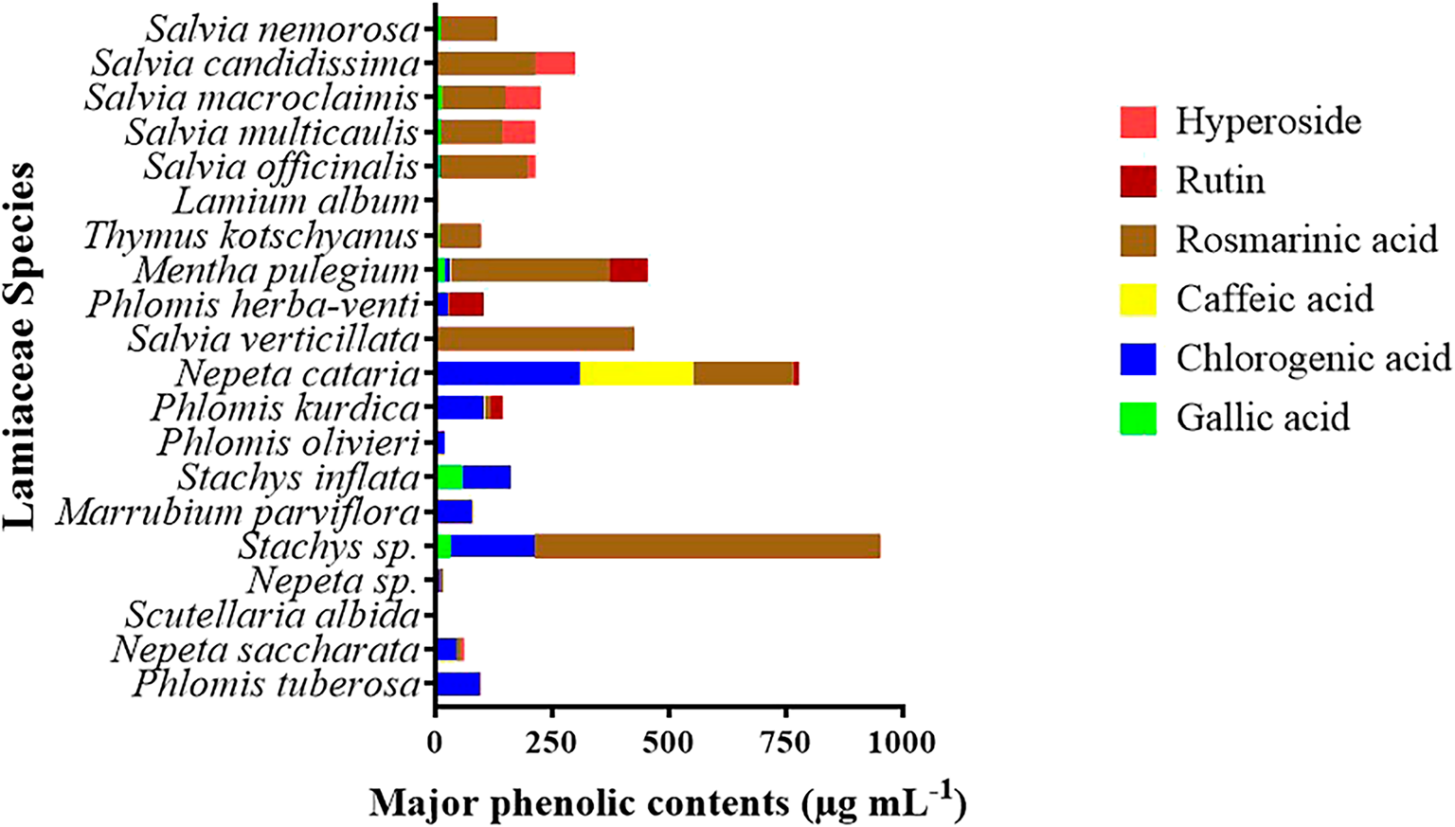
Major phenolic compounds present in the 20 species from the various genera within Lamiaceae identified by HPLC analysis.

The analysis of phenolic phytochemicals, including individual flavonoids and phenolic acids (such as rosmarinic acid, gallic acid, *p*-hydroxybenzoic acid, chlorogenic acid, caffeic acid, *p*-coumaric acid, ferulic acid, rutin, hyperoside, luteolin, apigenin), present in extracts from the 20 species of Lamiaceae family, was conducted by HPLC. The chromatogram of standards and the contents of major individual phenolics in the studied species are depicted in Figs. 2 and 3, respectively. It is evident that among the phenolics analyzed, phenolic acids exhibited the highest levels of content. This finding is in strong agreement with the results previously obtained for other *Salvia* species^27–29^. Specifically, as depicted in Fig. 3, the most abundant phenolics in the analyzed species were rosmarinic acid (740.13 µg mL^−1^), chlorogenic acid (308.33 µg mL^−1^), and caffeic acid (243.01 µg mL^−1^), followed by hyperoside (85.54 µg mL^−1^), rutin (81.30 µg mL^−1^), and gallic acid (57.54 µg mL^−1^). Notably, *Stachys* sp. exhibited the highest content of rosmarinic acid and chlorogenic acid, whereas *Nepeta cataria* stood out for its particularly abundant caffeic acid content. Instead, the highest hyperoside and rutin concentrations were observed for *Salvia candidissima* and *Mentha pulegium*, respectively (Fig. 3).

Previous studies on family Lamiaceae revealed the existence of polyphenolic compositions^30–33^. The main polyphenols characterizing this family are phenolic acids and flavonoids. There is a wide distribution of phenolic acids among the various genera within the Lamiaceae family, both in terms of their type and abundance. Among the most reported phenolic acid compounds are caffeic acid, *p*-hydroxybenzoic acid, caffeoyl tartronic acid, protocatechuic acid, chlorogenic acid, ferulic acid, gallic acid, rosmarinic acid, vanillic acid, *p*-coumaric acid, and benzoic acid^34–40^. Notably, chlorogenic acid and caffeic acid have been suggested as important chemotaxonomic markers for distinguishing different genera within the Lamiaceae family^36^. Different classes and structures of flavonoid components including flavanone, flavones, flavonols, and flavanols have been previously identified in the extracts of various Lamiaceae species^34,37^.

In general, most of the phenolic acids in genus *Salvia* originate almost exclusively from caffeic acid. Caffeic acid holds major biochemical roles within species of the Lamiaceae family and is frequently observed in its dimeric form (known as rosmarinic acid) in plants^41^. Rosmarinic acid is the primary component phenolic acid in *Salvia* species^42,43^, and it has also been identified in several other species of Lamiaceae family^44^. In a study by Munné-Bosch and Alegr, variations in the levels of rosmarinic and caffeic phenolic acids were documented across 96 different genera within the Lamiaceae family^45^. In accordance with their findings, our results indicate that concentrations of rosmarinic acid were notably higher than those of caffeic acid for all the investigated species, with the only exception of *Nepeta cataria*.

### Ascorbic acid content (AAC)

Statistically significant differences in AAC (*p* < 0.01) were observed among the studied samples. The AAC values ranged from 0.58 to 1.15 mg AA/ g DW (Table 3) with *Salvia officinalis* and *Stachys* sp. showing the highest and lowest ascorbic acid contents, respectively. In previous studies, the AAC varied between different species within the Lamiaceae family^46^. Differences in AAC could be attributed to genetics, climate, weather, and environmental conditions^47,48^. The biosynthesis and accumulation of AAC in various species are inherited^49^ and vary with environmental stimuli, such as light intensity, ethylene hormone, temperature and hypoxia^50^. Ascorbic acid acts as a donor of single hydrogen atoms to lipid radicals, leading to the decomposition of singlet oxygen and the removal of molecular oxygen^51^. Indeed, the biochemical activity of ascorbic acid is mainly related to its reduction potential^52,53^. In other words, ascorbic acid has the property of being easily oxidized in aqueous solution by releasing electrons, so it can function as a powerful antioxidant that reacts with reactive oxygen species (ROS) or free radicals^54^.

### Antioxidant activity (AOA)

The antioxidant properties of the plant extracts were assessed by DPPH and FRAP assays, with results presented in Table 3. These findings unveiled significant differences in the exhibited antioxidant activity (AOA) across the diverse studied species (*p* < 0. 01).

Particularly noteworthy were the results of *Salvia nemorosa* and *Salvia macrochlamys*, which showed the highest antioxidant capabilities in the DPPH (58.86 mg AAE/g DW) and FRAP (77.21 μmol Fe^++^/g DW) assays, respectively. In contrast, *Nepeta* sp. demonstrated the lowest antioxidant potential in both assays (26.67 mg AAE/g DW from DPPH and 3.76 μmol Fe^++^/g DW from FRAP), as detailed in Table 3.

Rich sources of antioxidants in Lamiaceae family belong to the Nepetoideae subfamily, including *Salvia*, *Mentha*, *Melissa*, etc. These plants contain some phenolic acids and are often rich in volatile aromatic terpenes^55^. In the study reported by Kaefer and Milner^56^, *Thymus vulgaris*, *Salvia officinalis*, *Rosmarinus officinalis*, and *Origanum majorana* showed the highest antioxidant properties among the investigated medicinal plants. Later, Albayrak et al.^57^, showed that *Thymus vulgaris*, *Salvia officinalis*, *Rosmarinus officinalis*, as well as *Mentha piperita*, *Melissa officinalis*, and *Ocimum basilicum* have a considerable content of phenols with potent total antioxidant and DPPH free radical scavenging properties. The above species are among the most studied species of the Lamiaceae family and their antioxidant capacity has been demonstrated in several other studies^58–63^. Polyphenols are naturally occurring phytochemicals found mostly in medicinal plants and often involved in defense against free radicals. Phenolics play a key role in absorbing and neutralizing free radicals, scavenging singlet oxygen produced by the triplet states, and decomposing peroxides. Polyphenolic compounds have been recognized for their robust antioxidant capabilities largely attributed to the positioning of hydroxyl groups within their structures. This is evident in the simultaneous presence of dihydroxyl and carboxyl groups in their aromatic rings^64^. Natural plant antioxidants can be classified into several main classes: phenolics (including flavonoids, anthocyanins, tannins, and phenolic acids), vitamins (such as tocopherols), ascorbic acid, and carotenoids^65^. Among these, phenolic acids (like rosmarinic, caffeic, chlorogenic, ferulic, *p*-coumaric, and vanillic acids) are known as natural antioxidants extensively present in medicinal plants.

### Pearson correlation coefficient analysis

**Figure 4:**
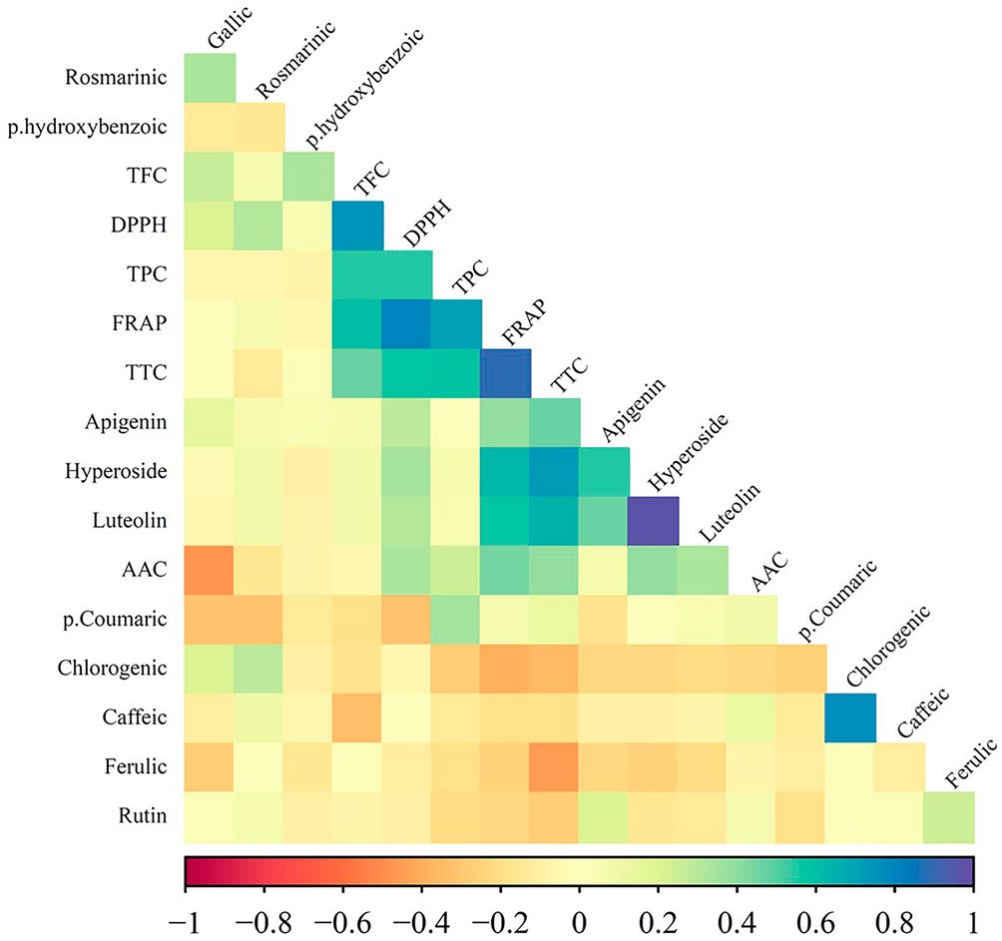
Heatmap of the Pearson correlation coefficient matrix for the traits studied across the 20 species of the Lamiaceae family. The abbreviations used are as follows: *TPC* Total Phenol Content, *TFC* Total Flavonoid Content, *AAC* Ascorbic Acid Content, *TTC* Total Tannin Content, *DPPH* antioxidant activity based on 2,2-diphenyl-1-picrylhydrazyl assay, *FRAP* antioxidant activity based on ferric reducing antioxidant power assay.

The results of the Pearson correlation coefficient analysis for the studied traits are presented in Fig. 4. Positive correlations are indicated in blue, while negative correlations are in red. The magnitude of the correlation coefficients is proportional to the intensities of the colors. The strongest positive correlation was observed between hyperoside and luteolin flavonoids (r = 0.98). This was followed by TTC and FRAP (r = 0.89), TFC and DPPH (r = 0.76), and chlorogenic acid and caffeic acid (r = 0.77) (Fig. 4). Also, TPC exhibited an intense positive correlation with the antioxidant activity determined by FRAP assay (r = 0.71). Therefore, from this analysis TTC and TFC clearly emerged as the main contributors to the observed antioxidant activities of samples (Fig. 4). These results also agree with existing studies in the literature, highlighting a strong positive relationship between antioxidant properties and phenolic compounds^66^. A study involving 11 *Salvia* species across Europe also revealed a strong positive correlation between the TPC and scavenger capacity^67^. Numerous investigations have consistently reported a significant correlation between antioxidant properties and polyphenolic compounds^68,69^. Several reports emphasize the connection between the antioxidant capacity of herbs and their content of phenolic components, including flavonoids, anthocyanins, phenolic acids, and tannins^70^.

### Lamiaceae family screening based on HCA and PCA

**Figure 5:**
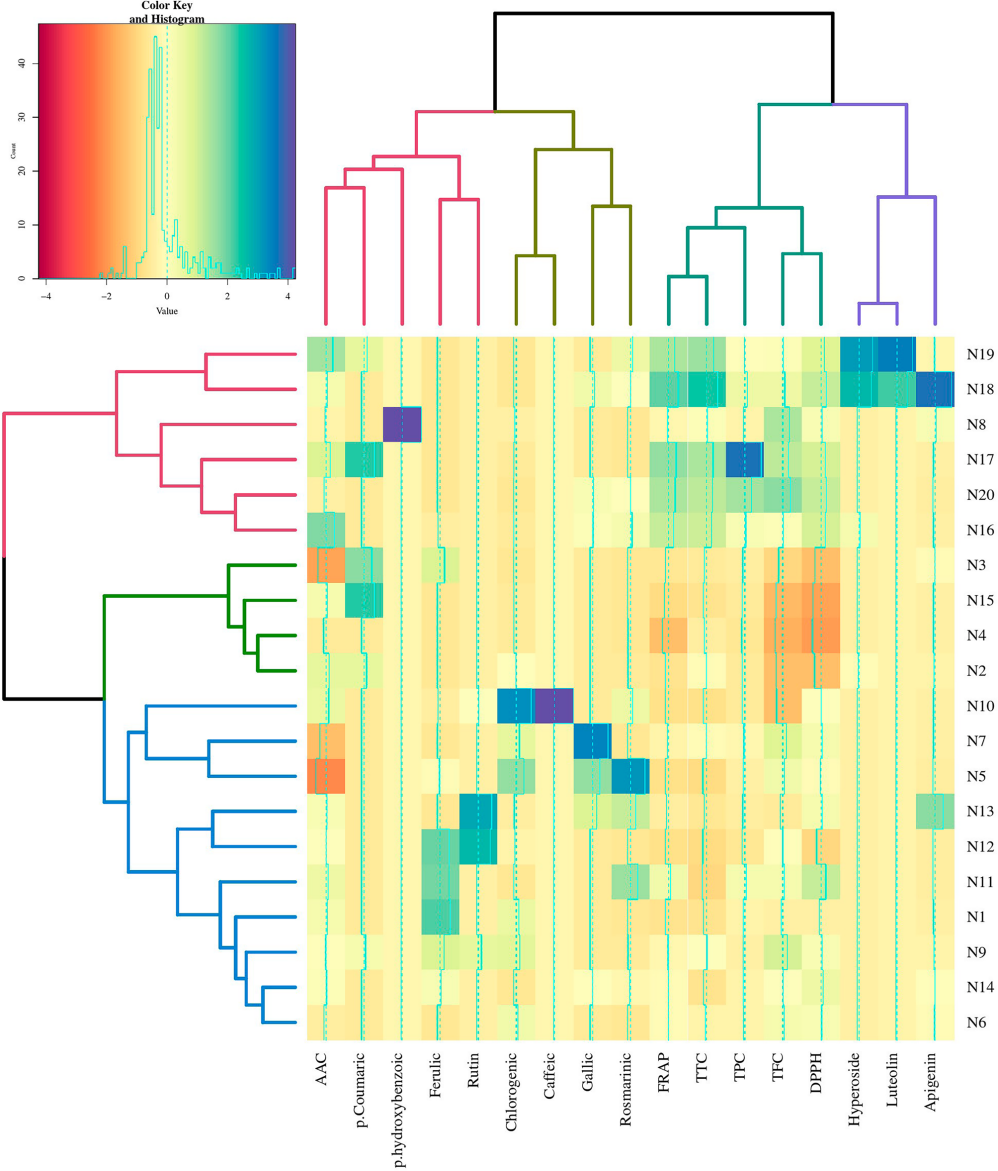
Dendrogram of hierarchical clustering analysis (HCA) and heatmap of phytochemical properties of species across various genera within the Lamiaceae family. The abbreviations used are as follows: *TPC* Total Phenol Content, *TFC* Total Flavonoid Content, *AAC* Ascorbic Acid Content, *TTC* Total Tannin Content, *DPPH* antioxidant activity based on 2,2-diphenyl-1-picrylhydrazyl assay, *FRAP* antioxidant activity based on ferric reducing antioxidant power assay.

A classification of the 20 investigated species of the Lamiaceae family was carried out through the Hierarchical Clustering Analysis (HCA), using the Euclidean distance and the Ward method, considering the 17 main properties (including phytochemical components and antioxidant capacity). Based on the HCA, the species within the Lamiaceae family were categorized into three distinct groups (Fig. 5). The first group comprised the *Salvia* species (*S. officinalis*, *S. multicaulis*, *S. macrochlamys*, *S. candidissima*, *S. nemorosa*) characterized by elevated levels of TPC, TFC, TTC, AAC, antioxidant capacity (measured via FRAP and DPPH assays), and various individual phenolic components. The second group included *Nepeta saccharata*, *Scutellaria albida*, *Nepeta* sp., and *Lamium album* species, which showed relatively low contents of phenolics and antioxidant capacity. The third group encompassed most of the species showing moderate levels of phytochemicals and antioxidant capacity, along with high levels of specific individual phenolic compounds.

**Figure 6:**
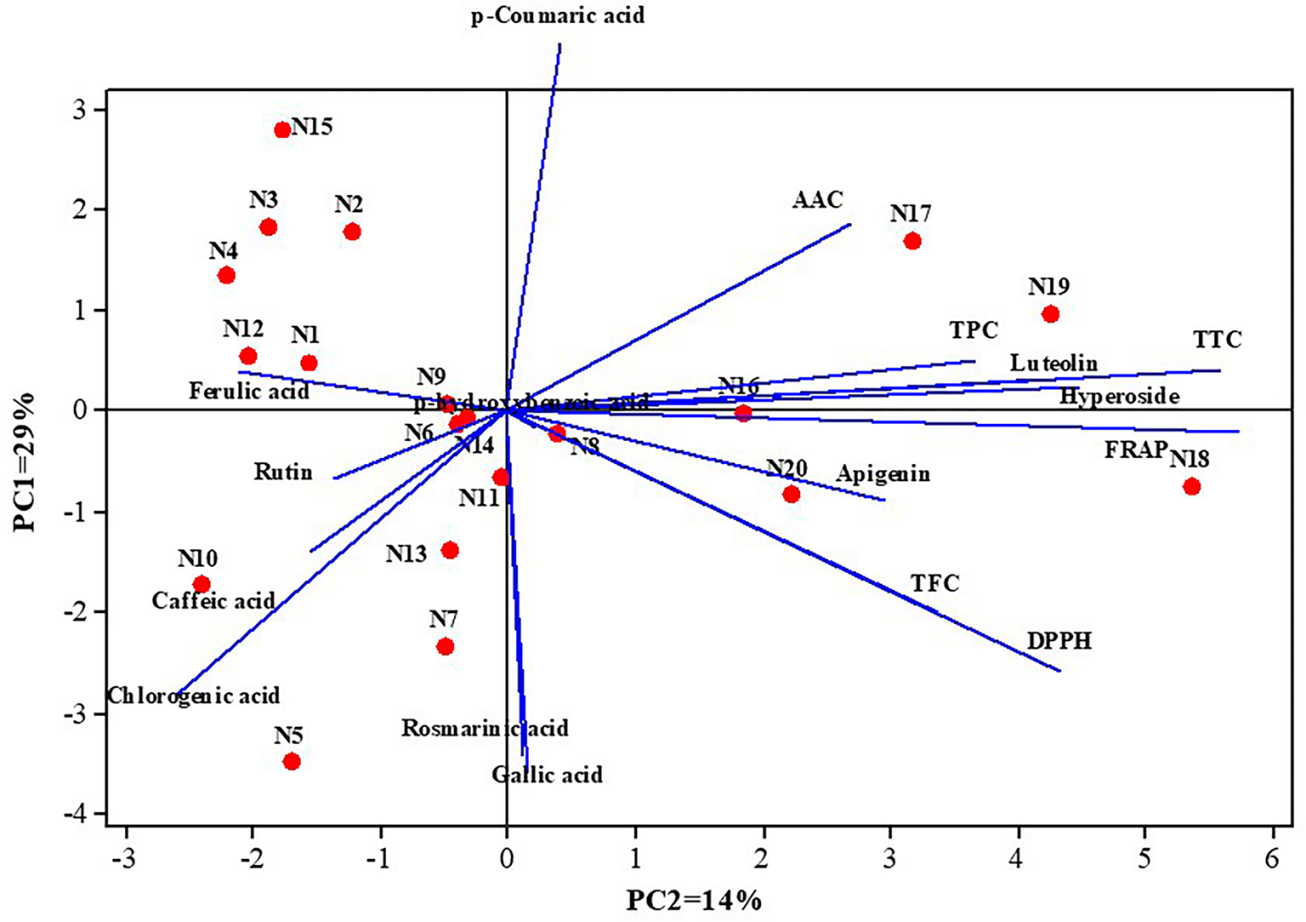
Biplot graph based on the principal components analysis (PCA) depicting the phytochemical properties across the 20 species of the Lamiaceae family. The abbreviations used are as follows: *TPC* Total Phenol Content, *TFC* Total Flavonoid Content, *AAC* Ascorbic Acid Content, *TTC* Total Tannin Content, *DPPH* antioxidant activity based on 2,2-diphenyl-1-picrylhydrazyl assay, *FRAP* antioxidant activity based on ferric reducing antioxidant power assay.

Finally, a PCA analysis was performed to further confirm the classification determined by HCA for the species within the Lamiaceae family (Fig. 6). PCA is an unsupervised statistical method that allows for the identification of patterns within a dataset, revealing hidden similarities and/or differences. Particularly, PCA was performed to identify associations between phytochemical compositions and antioxidant capacity among different species, as well as to classify them. The variation of the data explained was around 29% and 14% for principal component 1 (PC1) and principal component 2 (PC2), respectively (43% of the total variation). The PC1 showed a strong positive correlation with antioxidant capacity (by FRAP and DPPH assays), TTC and TPC, as well as hyperoside and luteolin flavonoids. The PC2 classified the Lamiaceae family species based on antioxidant activity (by DPPH assay), gallic acid, chlorogenic acid, rosmarinic acid. Also, PC2 had a strong negative correlation with *p*-coumaric acid.

## Conclusion

To explore the Lamiaceae family for potential sources of phytochemicals and antioxidants, a comprehensive evaluation was conducted on 20 species within this family. Among the species under investigation, those belonging to the *Salvia* genus exhibited higher levels of TPC, TFC, TTC, AAC, and antioxidant activity than other species. The most abundant phenolic compounds in the analyzed species extracts included rosmarinic acid, chlorogenic acid, and caffeic acid. According to the HCA and PCA, three groups of species are recognized. The *Salvia* species were placed in the first group (*S. officinalis*, *S. multicaulis*, *S. macrochlamys*, *S. candidissima*, *S. nemorosa*) with high levels of TPC, TFC, TTC, AAC, antioxidant capacity and some individual phenolic compounds. Overall, the results of the present study suggest that the investigated species (particularly those within the *Salvia* genus) possess high antioxidant activity and various phytochemicals. Moreover, the results show that most of these species possess multiple compounds with beneficial properties.

## Data Availability

The datasets used and/or analyzed during the current study available from the corresponding author on reasonable request.
